# The use of infrared technology as a novel approach for studies with female laboratory animals

**DOI:** 10.3325/cmj.2020.61.346

**Published:** 2020-08

**Authors:** Martina Ratko, Nikola Habek, Milan Kordić, Aleksandra Dugandžić

**Affiliations:** 1 Croatian Institute for Brain Research, University of Zagreb School of Medicine, Zagreb, Croatia; 2 Centre of Excellence for Basic, Clinical and Translational Neuroscience, University of Zagreb School of Medicine, Zagreb, Croatia; 3 Department of Physiology and Immunology, University of Zagreb School of Medicine, Zagreb, Croatia; 4 MKP Ltd., Zagreb, Croatia

## Abstract

**Aim:**

To determine the changes in skin temperature and brown adipose tissue (BAT) activity throughout the estrous cycle as well as the regularity of the estrous cycle in mice.

**Methods:**

We assessed the differences in the duration of the estrous cycle and its phases between 3- and 8-month-old female mice (n = 18). Skin temperature and BAT activity were measured by infrared technology and compared with human menstrual cycle.

**Results:**

Young and old female mice did not differ significantly in the estrous cycle length. However, young animals had longer diestrus and shorter proestrus phase. In contrast with women, mice showed age-dependent changes in body temperature and BAT activity during the estrus cycle.

**Conclusion:**

Establishing the pattern of temperature and BAT activity changes could be used to determine the estrous cycle phase before performing experiments without disturbing the animal. However, since the regulation of BAT activity during the estrous cycle was age-dependent, very complex, and varied significantly from women, further studies are needed to develop a non-invasive method for determining the phase of the estrous cycle.

It has become more and more evident that the representation of female subjects in animal studies should be increased. Funding organizations encourage studies in both male and female subjects, but it is very important that experiments in female population include the determination of the phases of the estrous cycle. Even though the number of studies involving female subjects increases, studies are badly planned, consequently yielding unreliable conclusions. If at the day of experiment, female mice are in the same phase of the estrous cycle, data will not vary significantly (1). However, a few days later the results could be completely opposite. Sometimes, when the phases of the estrous cycle are not considered, there is no difference between male and female animals, but the difference occurs when female animals are in estrous (E) compared with when they are in diestrus (DE). Furthermore, it is also problematic to perform behavior tests in male and female laboratory animals without the possibility to mimic the pathological conditions relevant to female population (1). This problem might be solved by determining the phase of the estrous cycle. Our recent study explained in detail how to correctly perform some behavior tests, and how the estrous cycle affects the collected data and expression of relevant proteins in the amygdala (2). The effects of estrogens and progesterone on cellular, organ, and body function are numerous, not to mention possible effects on pathological conditions and therapy during different phases of the estrous cycle. Estrogen regulates energy metabolism and glucose homeostasis (3), and impaired glucose tolerance was observed in proestrus (PE) compared with other phases of the estrous cycle in mice (4). Studies that assess the effects of sex hormones in a physiological condition during the phases of the estrous cycle in laboratory animals or in women during the follicular or luteal phase of the menstrual cycle are extremely rare. A study cannot merely involve the same number of female and male animals. In most cases at least four times more female than male animals are needed since the duration of the estrous cycle can significantly vary (5). In metabolic and behavior studies, determining the phase of the estrous cycle by vaginal smears just before conducting the experiments causes additional stress to animals, which makes it demanding to accurately predict the phase of the estrous cycle.

In humans, basal body temperature changes throughout the menstrual cycle. A day after ovulation the body temperature increases by up to 0.5 °C. The basal body temperature is affected by the activity of brown adipose tissue (BAT) (non-shivering thermogenesis). BAT activates in several physiological conditions: after cold exposure (thyroid hormones) (6-8), after a meal (through activation of hypothalamic guanylate cyclase C) (9), and during stress (sympathetic system) (10-12). BAT activity differs in men and women and decreases by aging (13). After menopause, the core temperature and BAT activity decrease (14). The mechanism behind the changes in core body temperature and BAT activity during the menstrual cycle is not completely understood. The majority of studies were performed on laboratory animals following bilateral ovariectomy, which does not reflect the physiological condition in these animals. Thermoregulation followed by changes in BAT activity can be affected by both estrogen and progesterone, both of which regulate BAT activity via the central nervous system or directly (15). Therefore, the aim of this study is to determine the changes in skin temperatures and BAT activity throughout the estrous cycle (and their similarity to the menstrual cycle), as well as the regularity of the estrous cycle in mice. We evaluated the feasibility of employing infrared technology in determining the estrous cycle phase in mice, which could prove a less stressful method for the animal than the currently employed method of vaginal smears.

## Materials and methods

### Animals

The experiments were carried out at the Department of Physiology, University of Zagreb School of Medicine, during summer time. Animals were 32.7 ± 1.8 (31.1-35.1, n = 12) week-old (correspond to 40-year-old women) and 14.4 ± 3.1 (11.6-17.1, n = 6) week-old (correspond to 25-year-old women) wild type (WT) C57Bl/6NCrl female mice. All animals were housed in the same room, under constant environmental conditions (temperature 24 °C, humidity 60%) and 12:12 light/dark cycle (6 am-6 pm). The experiments were performed between 10.00 and 12.00 am Mice were fed with standard rodent chow (GLP [PF1610] Mucedola srl, Settimo Milanese, Italy) *ad libitum* with free access to water. Before the experiments, the animals were confined in polystyrene cages, 4-6 per cage. The day before the experiments, the animals were separated in individual cages.

To determine BAT activity, the animals fasted for 4 hours before the experiments. The phase of the estrous cycle was determined after measuring BAT activity and temperature by staining of vaginal swabs with 0.1% cresyl violet as previously described (16). The smears were visualized with Olympus SZX10 stereomicroscope, and images were taken with AmScope MT1000 digital camera (United Scope LLC, Irvine, CA, USA).

All procedures were performed in accordance with the Ethical Codex of Croatian Society for Laboratory Animal Science and approved by the University of Zagreb School of Medicine Ethics Committee (EP 185/2018). The experiments were performed in accordance with the ARRIVE guidelines. All efforts were made to minimize animal suffering and to reduce the number of animals used.

### Infrared thermography

The temperature over interscapular BAT (iBAT) was measured for 10 consecutive days using a thermal camera (FLIR T-650sc, FLIR Systems, Wilsonville, OR, USA) after 4 h of fasting to avoid BAT activation after a meal. The researchers were “blinded” to the group of animals that was scanned since the estrous cycle was determined after the measurements were performed. The interscapular area of the animals was shaved the day before the experiment and additionally when necessary during the 10 days of the experiments. For measurements, each animal was placed in a clean polystyrene cage. Animals were allowed to adjust to new environment and filmed with thermal camera for 30 s. The camera software calculates the object’s temperature by taking into account the emissivity of mouse skin (e = 0.97), reflected room temperature (24.8 ± 0.8 °C), air temperature (24.2 ± 0.6 °C), relative humidity (60.2 ± 4.4%, n = 16), and distance to the object (1 m) as these values can affect the measurements. These values did not differ between the tested groups.

To determine BAT activity we calculated iBAT heat energy output in watts (W) with the Stefan-Boltzmann law (17):

BAT activity (W) = ϵ × σ × A × T^4^

where ϵ is emissivity of 0.97, σ is Stefan-Boltzmann’s constant (5.676 × 10^−8^ Wm^2^ K^4^), A is an area of the skin warmer than 34.6 °C (region of interest) in m^2^, and T is the mean temperature of the area in K.

### Statistical analysis

The data are expressed as median and interquartile range (IQR) or mean and standard deviation (SD). Normality of distribution was tested by the Shapiro-Wilk test. The significance of differences in the duration of the estrous cycle and its phases was tested by non-parametric Mann-Whitney U test. For other analyses, the *t* test and one-way ANOVA followed by the Tukey *post hoc* test were performed. The level of statistical significance was set at *P* < 0.05. Statistical analysis was performed with IBM SPSS, version 25 (IBM Corp, Armonk, NY, USA).

## Results

### Duration of the estrous cycle and its phases in C57Bl/6NCrl mice

At the first day of the experiment, two 8-month-old female mice were in PE, seven in E, one in metestrus (ME), and two in DE. An equal number of 3-month-old mice were in E and DE.

**Figure 1:**
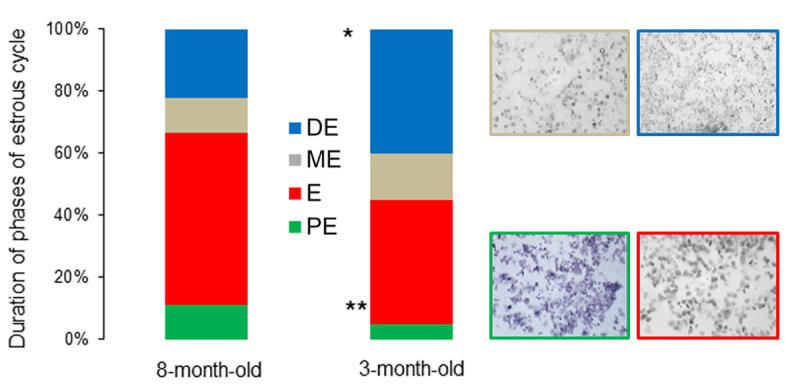
Duration of proestrus (PE – green), estrus (E – red), metestrus (ME – gray), and diestrus (DE – blue) in 10 days of observation. The original images of vaginal smears are shown on the left. Even though the length of the cycle did not differ between young (n = 6) and old female mice (n = 12), DE was significantly shorter, and PE significantly longer in old mice. *P = 0.041 significant difference between young and old female mice in the duration of DE. **P = 0.024 significant difference between young and old female mice in the duration of PE. The data were analyzed with the Mann-Whitney test. The results are shown as the percentage of duration of each phase of the estrous cycle in 10 days.

The duration of estrous cycle did not differ significantly between the groups (8-month-old mice: median [IQR] = 6 [2] days, n = 12; 3-month-old mice: 8 [3] days, n = 6, Mann-Whitney test, *P* = 0.3713). In older mice DE was significantly shorter (old mice: median = 2 [1] day, n = 12; young mice: 4 [2.25] days, n = 6, Mann-Whitney test, *P* = 0.041) and PE significantly longer (old mice: 1 [1] day, n = 12; young mice: 0.5 [1], n = 6, Mann-Whitney test, *P* = 0.024) (Figure 1).

### Changes in skin temperature and BAT activity during the estrous cycle

**Figure 2:**
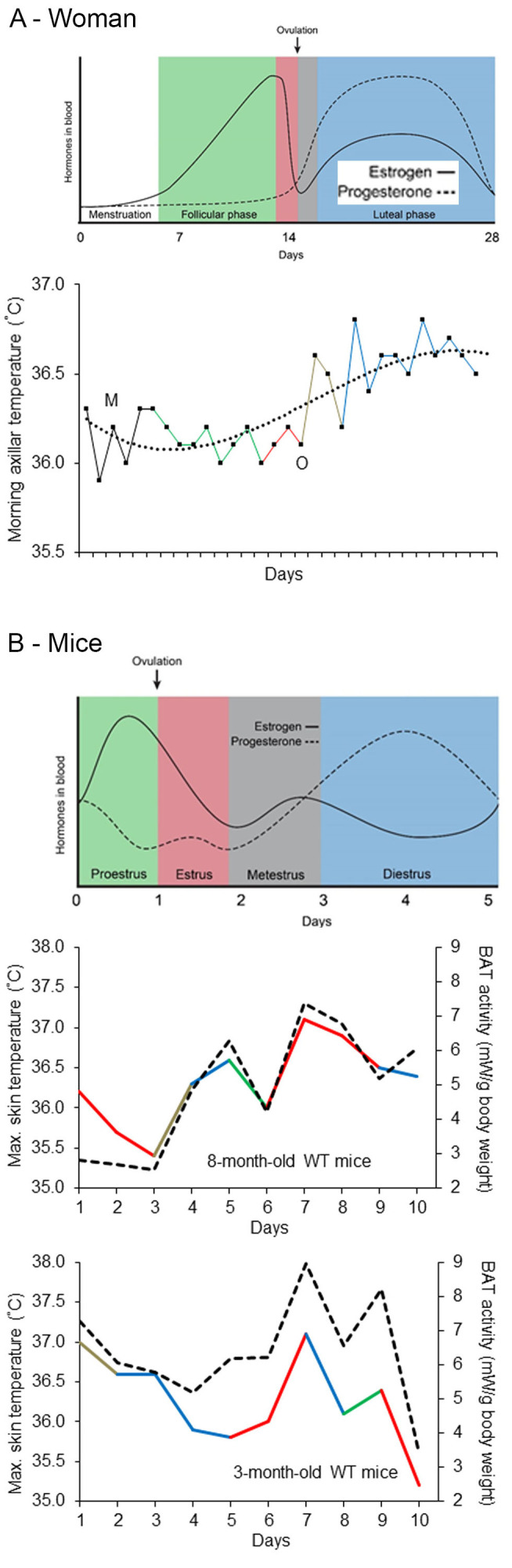
Original data of changes in skin temperature and brown adipose tissue (BAT) activity during the menstrual cycle in a woman and the estrous cycle in 3- and 8-month-old wild-type (WT) mice. The morning axillar body temperature (**A**) increases after ovulation (O) (dotted line). Skin temperature and BAT activity differ between 3- and 8-month-old WT mice (**B**). Proestrus (PE – green), estrus (E – red), metestrus (ME – gray), diestrus (DE – blue), menstruation (M – black). Upper panels are schematics of estrogen and progesterone changes during the menstrual and estrous cycle (modified from: 18).

To compare the estrous cycle in mice with the menstrual cycle in women, the morning axillar body temperature in a female volunteer was measured. As expected, body temperature changed biphasically, showing an increase of 0.45 °C in the postovulatory period (follicular phase [PE]: 36.14 ± 0.13 °C, n = 14; luteal phase [ME and DE]: 36.57 ± 0.16 °C, n = 13, paired t (12) = 6.489 *P* = 0.00003), which corresponds to an increase in prostaglandin secretion (Figure 2). The luteal phase in the mouse is divided in ME and DE. During ME, the corpus luteum starts to produce progesterone (modified from 18, upper panel Figure 2A). Therefore, the female menstrual cycle was artificially divided to correspond to the estrous phases in mice (upper panel Figure 2B).

**Figure 3:**
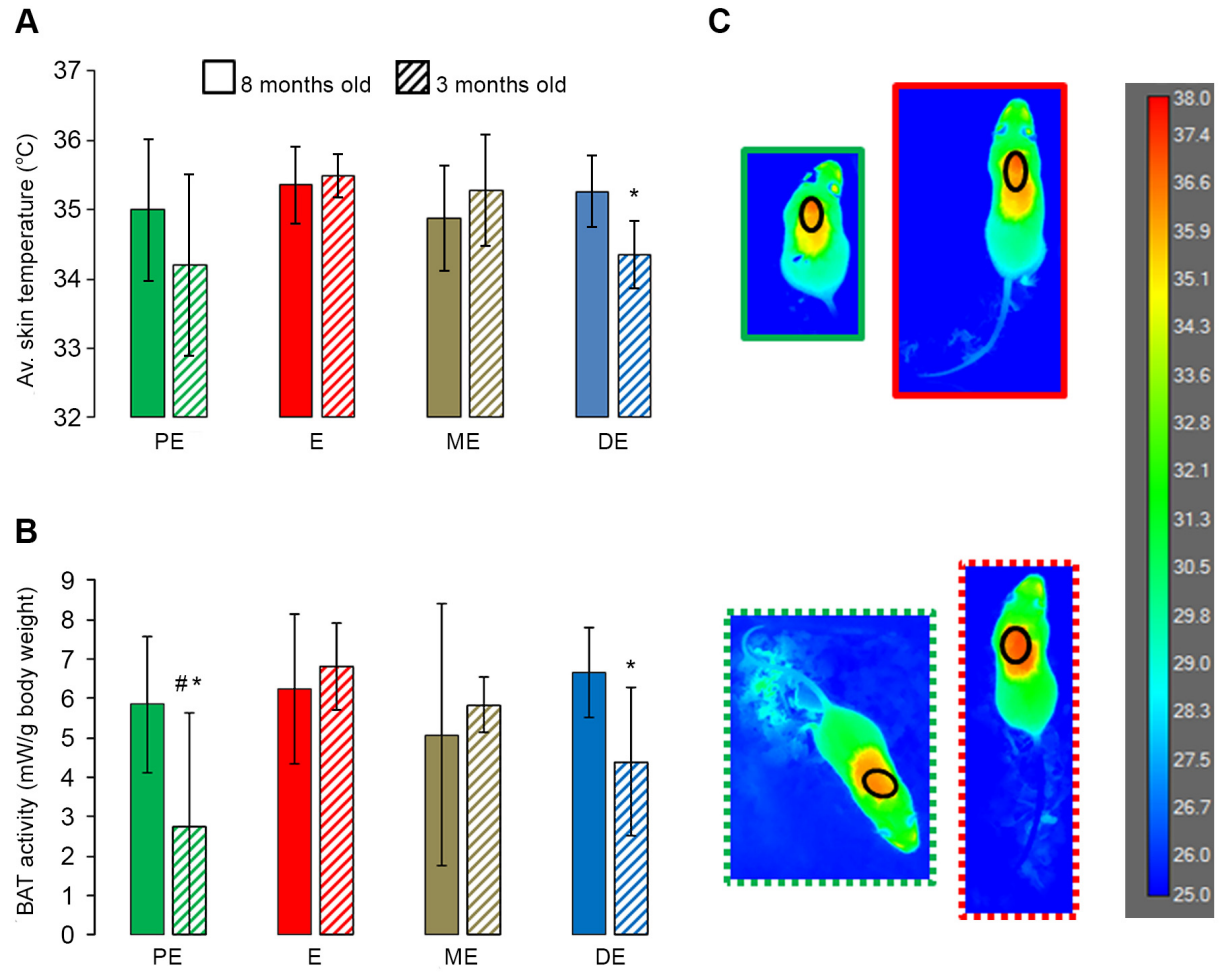
Summarized changes of skin temperature (**A**) and brown adipose tissue (BAT) activity (**B**) during the estrous cycle in 3- (n = 5) and 8-month-old mice (n = 12). Skin temperature is presented as the average skin temperature of the encircled BAT area shown on the original scans on the right. *Compared with old mice, unpaired *t* test: t (14) = 3.28, *P* = 0.005. BAT was less active in PE compared with E in young mice (# – ANOVA: F(3,13) = 4.169, *P* = 0.028, *post hoc* Tukey test *P* = 0.026, n = 5). When compared with old mice (n = 12), young mice (n = 5) had lower activity during PE and DE (*t-test PE: t (12) = 2.404, *P* = 0.033, n = 12; DE:t (14) = 3.017 *P* = 0.009, n = 5). (**C**) Original paired scans with encircled interscapular BAT area showing decreased skin temperature and BAT activity in young mice (dotted lines) in PE and E. Results are shown as mean ± SD. PE – proestrus, E – estrus, ME – metestrus, DE – diestrus.

In 8-month-old female mice, skin temperature increased during ME and DE (Figure 2B color coded with gray and blue) and again during E (red), which corresponds to an increase in progesterone and estrogen secretion. In 3-month-old female mice, temperature increased during E, corresponding to estrogen production (Figure 2). The changes in BAT activity corresponded to changes in temperature (summarized at Figure 3).

### Predicting the diestrus phase via changes of skin temperature and BAT activity

Since we confirmed significant variations in the duration of different phases of the estrous cycle, the question is whether they could be detected without disturbing the animal by measuring its skin temperature or BAT activity. Older animals exhibited no significant difference in skin temperature between the phases of the estrous cycle (average temperature for each phase during 10 days, ANOVA: F(3,40) = 0.945, *P* = 0.428, n = 12). In young animals there was a difference between the phases of the estrous cycle (ANOVA: F(3,13) = 3.457, *P* = 0.048, n = 5). However, the *post hoc* Tukey test showed no significant difference. Skin temperature in DE was significantly lower in young than in old animals (old: 35.3 ± 0.5 °C, n = 12; young: 34.3 ± 0.5 °C, n = 5, unpaired t (14) = 3.28, *P* = 0.005). Therefore, it is not surprising that at the first day of DE in older mice the average skin temperature increased for 0.58 ± 0.76 °C (n = 12, significant effect: t (11) = 2.551, *P* = 0.0269), which is equivalent to body temperature change of women in the luteal phase. In younger animals, the skin temperature did not change significantly (0.46 ± 1.39 °C, n = 5, t(4) = 0.7456, *P* = 0.4973).

Changes in BAT activity were more pronounced and preceded the changes in temperature. In 8-month-old mice, BAT activity did not change during the estrous cycle (ANOVA: F(3,38) = 0.983, *P* = 0.411, n = 12), while in 3-month-old mice, BAT activity decreased in PE compared with E (ANOVA: F(3,13) = 4.169, *P* = 0.028, with *post hoc* Tukey test *P* = 0.026 between PE and E, n = 5). Younger mice had lower activity during PE and DE (*t* test PE: t (12) = 2.404, *P* = 0.033; DE:t (14) = 3.017 *P* = 0.009) (Figure 3). It is not surprising that on the first day of DE, BAT activity significantly increased in old mice (for 1.86 ± 2.08 mW/g body weight, n = 12, t (10) = 3.068, *P* = 0.0119), while this was not the case in young mice (-0.37 ± 2.35mW/g body weight, n = 5, t(4) = 0.3566, *P* = 0.7394).

## Discussion

In this study, as reported previously for other laboratory animals (5), C57Bl/6NCrl female mice had widely ranging estrous cycle duration. The estrous cycle phases did not last a day each (except for DE) as it is commonly considered (18), and their duration significantly differed between 3- and 8-month-old mice.

Lately, the scientific community has been urged to perform studies not only on male but also on female laboratory animals. These studies should be performed after careful planning. Performing experiments just to meet a requirement yields misleading conclusions and scientifically meaningless results. It is especially important that female animals are in the same phase of their estrous cycles because of a different effect of estrogen and progesterone on numerous physiological and pathological conditions (2,4).

Metabolic or behavioral studies should be performed without disturbing or stressing the animals. The irregularity of the estrous cycle makes it impossible to “plan” in which phase the female mice would be at the day of the experiment. As the changes in body temperature, which followed changes in BAT activity, are straightforward in humans, the idea was to use infrared technology to measure animal temperature and BAT activity without disturbing the animals.

Body temperature regulation during the menstrual cycle in women is still not completely understood. Estrogen and progesterone could affect body temperature through several mechanisms: by regulating different temperature set points in the hypothalamus, by regulating non-shivering thermogenesis by activating BAT, and by releasing the heat via vasodilatation in the skin (15,19).

BAT activity could be increased by estrogens centrally or peripherally. A study on ovariectomized rats (no age was mentioned) showed that estrogens via ventromedial hypothalamic and arcuate nucleus increased sympathetic and, therefore, BAT activity (3,20). Even though the estrogen receptors are found on the cells of adult human BAT as well (21), a direct effect of estrogen on human BAT remains unclear (14).

Studies of estrogen effects on BAT physiology conducted in animals without regard to the phase of the estrous cycle have another problem. For example, authors determined the expression of estrogen receptors in the brainstem (22) without separating the female rats by estrous phase, although receptors expression changes during the estrous cycle (23). Our previous study also showed a difference in expression regulation of guanylate cyclase C in the amygdala and hypothalamus during the estrous cycle (2). Results such as these emphasize the importance of determining the phase of the estrous cycle in any study involving female animals. It is obvious that, at least in the human population, the effects of estrogen on body temperature and BAT activity (proliferative phase) are controversial (14).

Considering the increase in body temperature during the luteal phase, it is not surprising that BAT also has progesterone receptors (24). It is suggested that progesterone also activates BAT by enhancing sympathetic stimulation (25).

Taking everything into consideration, it is not surprising that BAT activity in 8-month-old mice was high in PE, E, and DE, when it could be affected by both estrogen and progesterone. In younger mice, BAT activity was increased in E, since in these mice estrogen plays a more important role, contrary to findings in the human population (26). The original data show changes in BAT activity and body temperature for each day and they slightly differ from summarized results, which were averaged for each phase of the estrous cycle. A limitation of this study is that PE in some mice lasted less than a day. Since mice fasted 4 h before measurements, and re-feeding could affect the results, the measurements could not be performed twice a day.

Most studies that considered the estrous cycle phase used animals in DE even though this phase is characterized by highly active progesterone, a fact that is usually neglected. The difference between age groups in our study was more pronounced when we monitored changes in body temperature and BAT activity a day before DE, which could be used for the estimation of DE phase, since BAT activity increases on the first day of DE in older animals (similar to post-ovulatory increase in body temperature in women) with no changes in younger animals.

In summary, the physiological regulation of BAT during the menstrual cycle is still not well understood for several reasons. First, the studies on female laboratory animals are performed poorly, either on ovariectomized animals or without determining the phase of the estrous cycle. Second, the understanding of the physiological regulation of BAT activity during the human menstrual cycle is still basic. Taking into account the significant differences in BAT activity presented here, we cannot simply apply the results obtained in laboratory animals to humans. A better understanding of the influence of the phases of the estrous cycle and age on the changes in body temperature and BAT activity could lead to the development of a novel, non-invasive method for determining the phase of the estrous cycle before any experiments are performed.
